# Effect of endometriosis on the fecal bacteriota composition of mice during the acute phase of lesion formation

**DOI:** 10.1371/journal.pone.0226835

**Published:** 2019-12-30

**Authors:** Josefine Hantschel, Severin Weis, Karl-Herbert Schäfer, Michael D. Menger, Matthias Kohl, Markus Egert, Matthias W. Laschke

**Affiliations:** 1 Institute for Clinical & Experimental Surgery, Saarland University, Homburg/Saar, Germany; 2 Faculty of Medical and Life Sciences, Institute of Precision Medicine, Microbiology and Hygiene Group, Furtwangen University, Villingen-Schwenningen, Germany; 3 Campus Zweibrücken, University of Applied Sciences Kaiserslautern, Zweibrücken, Germany; 4 Faculty of Medical and Life Sciences, Institute of Precision Medicine, Group for Statistics in Biology and Medicine, Furtwangen University, Villingen-Schwenningen, Germany; University of Illinois, UNITED STATES

## Abstract

Accumulating evidence indicates that there is an interaction between the gut microbiota and endometriotic lesions. The new formation of these lesions is associated with stem cell recruitment, angiogenesis and inflammation, which may affect the composition of the gut microbiota. To test this hypothesis, we herein induced endometriotic lesions by transplantation of uterine tissue fragments from green fluorescent protein (GFP)^+^ donor mice into the peritoneal cavity of GFP^-^ C57BL/6 wild-type mice. Sham-transplanted animals served as controls. Fecal pellets of the animals were collected 3 days before as well as 7 and 21 days after the induction of endometriosis to analyze the composition of the gut microbiota by means of 16S ribosomal RNA gene sequencing. The transplantation of uterine tissue fragments resulted in the establishment of endometriotic lesions in all analyzed mice. These lesions exhibited a typical histomorphology with endometrial glands surrounded by a vascularized stroma. Due to their bright GFP signal, they could be easily differentiated from the surrounding GFP^-^ host tissue. Bacterial 16S rRNA genes were successfully PCR-amplified from the DNA extracts of all obtained mice fecal samples. However, no significant effect of endometriosis induction on the composition of the bacterial microbiota was detected with our experimental setup. Our findings allow careful speculation that endometriosis in mice does not induce pronounced dysbiosis during the acute phase of lesion formation.

## Introduction

Endometriosis, i.e. the presence of ectopic endometrial-like tissue outside the uterine cavity, is a painful gynecological condition, which affects numerous women of reproductive age worldwide [1]. Moreover, current treatment options are restricted to symptomatic pharmacological and surgical therapies without eliminating the cause of illness [2, 3], the latter still remaining unknown. This can be explained by the fact that endometriosis is a multifactorial disease, which is determined by the complex interaction of multiple genetic, hormonal, immunologic and environmental factors [4, 5].

The pathogenesis of endometriosis is characterized by the estrogen-driven formation of endometriotic lesions in the peritoneal cavity. According to the implantation theory, these lesions originate from retrogradely menstruated endometrial tissue [6]. The endometrial tissue implantation is associated with a strong activation of the immune system, stimulating peritoneal macrophages, neutrophils, mast cells and T cells to secrete pro-inflammatory cytokines and angiogenic growth factors into the peritoneal fluid [7, 8]. In addition, circulating stem cells and endothelial progenitor cells are recruited into newly developing endometriotic lesions, where they contribute to tissue formation and vascularization [9, 10].

Accumulating evidence suggests that the gut microbiota may be crucially involved in all these hormone-related, inflammatory, angiogenic and vasculogenic processes. In fact, the microorganisms within the gastrointestinal tract are not only essential for the digestion of food, but also contribute to the regulation of estrogen metabolism [11], systemic inflammation [12] and stem cell homeostasis [13]. Accordingly, we have developed the hypothesis that the gut microbiota may closely interact with endometriotic lesions and, thus, represent a key player in the pathogenesis of the disease [14].

Recently, our novel hypothesis has been supported by first preclinical studies in mice. Chadchan et al. [15] reported that altering the gut microbiota with antibiotic treatment reduces the growth of mouse endometriotic lesions, which were surgically induced by uterine tissue transplantation into the peritoneal cavity. By means of high-throughput DNA sequencing technology they further found that mice developed an altered composition of their gut microbiota 21 days after endometriosis induction, also referred to as dysbiosis. In contrast, Yuan et al. [16] detected such dysbiotic changes only after a rather long observation period of 42 days. This is a surprising finding, because uterine tissue grafts particularly promote a strong host tissue reaction during the first 7 days of engraftment [17].

The discrepancy between these findings highlights the fact that there is no standard protocol for microbiome analyses, which clearly complicates direct comparisons of different studies. Indeed, individual studies may differ in many methodological aspects, such as the used databases for species identification as well as the techniques of DNA extraction, PCR amplification of the 16S rRNA genes, sequence data processing and statistical analysis. Hence, it is important to bring more clarity in this young field of research by increasing the amount of available data on microbiota-endometriosis interactions. For this purpose, we performed 16S ribosomal RNA gene sequencing to compare the gut microbial composition in mice shortly before and after the induction of endometriosis by transplantation of uterine tissue fragments into the peritoneal cavity. Sham-transplanted animals served as controls. In contrast to the aforementioned studies [15, 16], we used the regularly updated SILVA database for species identification. Moreover, we applied more strict conditions for statistical testing.

## Materials and methods

### Animals

We used 12- to 16-week-old female C57BL/6 wild-type mice and transgenic C57BL/6-TgN(ACTB-EGFP)1Osb/J donor mice (Institute for Clinical & Experimental Surgery, Saarland University, Homburg/Saar, Germany) with a body weight of 20–25 g. They were housed in a temperature and humidity controlled facility with a 12-h light-dark cycle in high-pressure steam sterilization-treated individually ventilated cages, which were changed weekly to maintain a stable environment. The mice were housed in groups of 4 animals per cage. Each cage contained 2 endometriosis mice and 2 sham-transplanted controls to exclude the cage environment as a potential bias. The animals had free access to tap water and standard pellet food (Altromin, Lage, Germany).

All experiments were performed according to the German legislation on protection of animals and the National Institutes of Health Guide for the Care and Use of Laboratory Animals (Institute of Laboratory Animal Resources, National Research Council, Washington DC, USA) and were approved by the local governmental animal protection committee (Landesamt für Verbraucherschutz, Saarbrücken, Germany; permission number: 01/2016).

### Cycle control

The estrous stage of the mice was assessed by vaginal lavage. For this purpose, 15 μL of physiological saline solution were carefully pipetted into the vagina. The suspension was transferred on a glass slide and analyzed under a phase contrast microscope (CH-2; Olympus, Hamburg, Germany). To exclude relevant differences in steroid hormone levels between individual animals, only donor and recipient mice in the stage of estrus were included in the study at the beginning of the experiments, i.e. 3 days before endometriosis induction.

### Endometriosis model

For the induction of endometriotic lesions, we freely transplanted uterine tissue fragments from 4 C57BL/6-TgN(ACTB-EGFP)1Osb/J donor mice, in which all cells except red blood cells and hair express green fluorescent protein (GFP) [18], into the peritoneal cavity of 8 C57BL/6 wild-type mice.

The donor mice were anesthetized by an intraperitoneal (i.p.) injection of 75 mg/kg ketamine (Ursotamin^®^; Serumwerke Bernburg, Bernburg, Germany) and 15 mg/kg xylazine (Rompun^®^; Bayer, Leverkusen, Germany). After midline laparotomy, the uterine horns were carefully excised and transferred to a Petri dish containing warm physiological saline solution. The horns were opened longitudinally and uterine tissue fragments with a standardized size of 2 mm in diameter were removed using a dermal biopsy punch (Stiefel Laboratorium, Offenbach am Main, Germany). The donor animals were finally sacrificed by cervical dislocation.

The recipient animals were anesthetized with 2% isoflurane in oxygen and fixed in supine position under a stereomicroscope (Leica, Wetzlar, Germany). The abdomen was opened via a 3 mm midline incision and 10 GFP^+^ uterine tissue fragments in 400 μL sterile physiological saline solution were intraperitoneally injected into each mouse with a 1 mL syringe. Subsequently, the incision was closed with a 6–0 Prolene suture (Ethicon Products, Norderstedt, Germany) and topical skin adhesive (Histoacryl^®^; B. Braun Melsungen, Melsungen, Germany). Eight additional mice received only an intraperitoneal injection of 400 μL physiological saline solution without uterine tissue fragments. These sham-transplanted animals served as controls. Due to the robust immune system of rodents it was not necessary to administer antibiotics after surgery to avoid an infection.

At the end of the experiments, i.e. 21 days after the induction of endometriosis, the animals were anesthetized by i.p. injection of ketamine and xylazine as described above. After midline laparotomy, they were positioned under a Zeiss Axiotech microscope (Zeiss, Oberkochen, Germany) with a 100 W mercury lamp and filter for blue light epi-illumination. By this, GFP^+^ uterine tissue fragments could easily be detected and distinguished from the surrounding GFP^-^ tissue of the recipient animals. The take rate of uterine tissue fragments (%), which had successfully engrafted and developed into endometriotic lesions per animal, was assessed. Moreover, the largest (D1) and perpendicularly aligned smallest diameters (D2) of the lesions were measured by means of a digital caliper. The lesion sizes (mm^2^) were then calculated with the formula D1 x D2 x π/4 [19]. The values were expressed as mean ± standard error of the mean.

Subsequently, the animals were sacrificed by cervical dislocation and the lesions were excised and fixed in formalin. After embedding in paraffin, the tissue samples were cut in 3-μm thick sections and stained with hematoxylin and eosin (HE) according to standard procedures to examine their morphology under a BX60 microscope (Olympus).

### Feces collection

**Fig 1:**
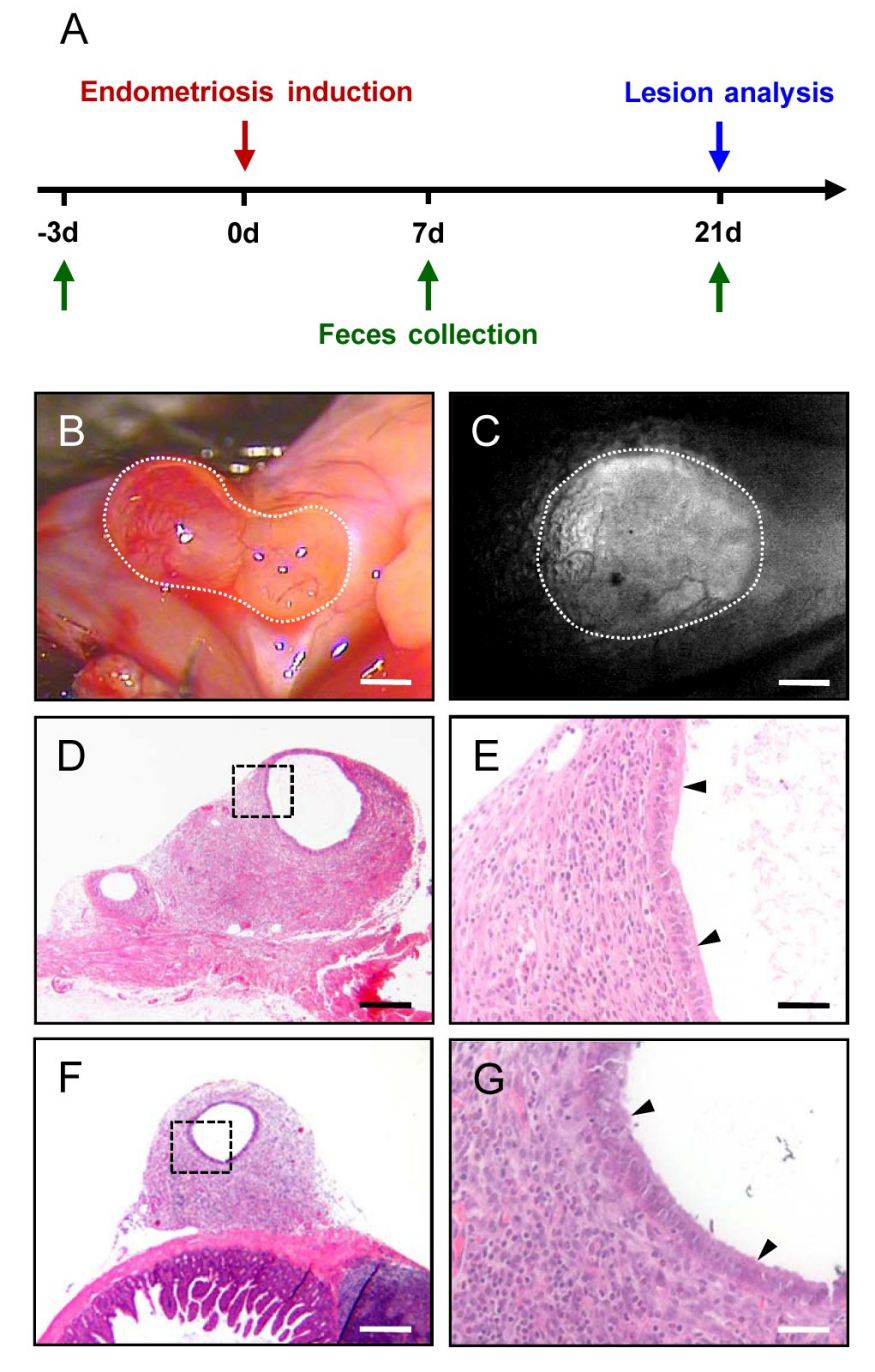
Endometriosis model. A: Sequence of the in vivo experiments. Endometriosis was induced on day 0 by transplantation of uterine tissue fragments from GFP^+^ donor animals into the peritoneal cavity of GFP^-^ recipient mice. After 21 days, the take rate and size of newly formed endometriotic lesions were assessed and the lesions were analyzed by histology. For the analysis of the gut microbiota, fecal samples were collected 3 days before the transplantation of uterine tissue fragments as well as on day 7 and 21 after the induction of endometriosis. B: Macroscopic appearance of a well vascularized endometriotic lesion (borders marked by dotted line) at the abdominal wall of a C57BL/6 mouse on day 21. Scale bar = 650 μm. C: Fluorescence microscopy of a newly formed endometriotic lesion in the mesentery on day 21. Due to its bright GFP signal, the lesion (borders marked with dotted line) can be easily detected in blue light epi-illumination microscopy within the surrounding GFP^-^ host tissue. Scale bar = 470 μm. D-F: HE-stained sections of endometriotic lesions at the abdominal wall (D, E) and the gut wall (F, G). Higher magnification (E, G = inserts in D, F) reveals a typical histomorphology of the lesions, i.e. cyst-like dilated glands with an epithelial layer (arrowheads), which are surrounded by a well vascularized endometrial stroma. Scale bars: D, F = 290 μm; E, G = 45 μm.

For the analysis of the gut microbiota, fecal pellets of endometriosis and control mice were collected 3 days before the transplantation of uterine tissue fragments as well as on day 7 and 21 after the induction of endometriosis (Fig 1A). For this purpose, each mouse was kept in a sterilized bedding-free cage to avoid potential cross contamination. The fecal pellets were collected in sterile freezing tubes, which were immediately transferred into liquid nitrogen and finally stored at -80°C until further analysis.

### DNA isolation

DNA isolation was performed using the ZymoBIOMICS DNA Miniprep-Kit (Zymo Research Europe, Freiburg, Germany) using 45–90 mg of well homogenized fecal material following the supplier´s instructions. For an optimal cell lysis, the extraction protocol included a 1 min bead beating step (repeated 5x, each) using a Fastprep-24 machine (MP Biomedicals, Eschwege, Germany) and bashing beads in lysis solution provided with the extraction kit. DNA purity and concentration after extraction were measured with an Implen NanoPhotometer P-Class 360 (Implen, Munich, Germany).

### Library preparation and sequencing

Sequencing library preparation of the V4 and V5 region of bacterial 16S rRNA genes was performed according to Illumina’s 16S Metagenomic Sequencing Library Preparation guide (Illumina, Eindhoven, Netherlands). The 16S-specific primers 520F (5’-CCGTCAATTCMTTTRAGTTT-3’) and 926R (5’-CCGTCAATTCMTTTRAGTTT-3’) were used to produce amplicons [20, 21]. Overhang adaptor sequence tails were added to address the Illumina indexing and binding systems (5’-TCGTCGGCAGCGTCAGATGTGTATAAGAGACAG-3’ to the 520F end and 5’-GTCTCGTGGGCTCGGAGATGTGTATAAGAGACAG-3’ to the 926R end). The isolated DNA served as template for two independent amplifications per sample. The PCR mixture consisted of 0.5 μL of each primer (Integrated DNA Technologies, Leuven, Belgium) (10 μM), 0.6 μL of dNTP-Mix (10 mM, each), 5 μL 5x KAPA Hifi Puffer including 20 mM MgCl_2_, 0.1 μL KAPA Hifi Polymerase (Roche, Mannheim, Germany), 1 μL DNA template and was filled up to a final volume of 25 μL with nuclease free water. PCR reactions were performed in a T100 Thermal Cycler (Bio-Rad Laboratories, Munich, Germany) using the following thermal profile: 3 min at 95°C for initial denaturation, 25 cycles of 30 s at 95°C for denaturation, 30 s at 55°C for annealing and 45 s at 72°C for elongation, followed by a final elongation step for 5 min at 72°C. Water-template controls and *Escherichia coli* DNA as positive controls were included for each set of the PCR reaction. Success of PCRs was verified by agarose gel electrophoresis using Midori Green as DNA-dye (Biozym, Olderndorf, Germany). Replicate PCRs of the same sample per sequencing set up were pooled and purified with Agencourt AMPure beads (Beckman Coulter, Krefeld, Germany) into 50 μL of 10 mM Tris (pH 8.5) buffer.

Subsequently, a second PCR step was performed to add unique index barcodes with sequencing adaptors to the amplicon targets. The index PCR reaction included 5 μL of Nextera XT (Illumina) Index Primer 1 and 5 μL of Nextera XT Index Primer 2 with 1.2 μL of dNTP-Mix (10 mM each), 10 μL 5x KAPA Hifi Puffer including 20 mM MgCl_2_, 0.2 μL KAPA Hifi Polymerase (Roche), 5 μL amplicon DNA and was filled up to 50 μL with nuclease free water. PCR reactions were performed in a T100 Thermal Cycler (Bio-Rad Laboratories) using the program detailed above, albeit with 8 cycles. The primers used targeted the V4 and V5 variable region of the bacterial 16S rRNA gene and led to amplicons of ~364 bp length, which is regarded sufficient for identification at genus level [22]. With indices and linker sequences, Illumina libraries had a mean sequence length of 528 bp. After purification with AMPure beads, quality checks for library sizes and DNA concentration were performed with the Agilent Bioanalyzer using Agilent DNA 1000 chips (Agilent Technologies, Waldbronn, Germany). The Qubit dsDNA HS Assay Kit (Thermo Fisher Scientific, Schwerte, Germany) was used to determine the DNA concentration. Finally, libraries of indexed amplicons for each sample were normalized to a concentration of 4 nM and pooled for sequencing.

The pooled libraries were sequenced on an Illumina MiSeq platform (Illumina) in a final concentration of 6 pM with 20% phiX control added, using the MiSeq Reagent Kit v3 in a 600-cycle (2x 300 bp + 2x 8 bp Index cycles) format following the manufacturer’s instructions.

### Bioinformatics

Sequence data were processed using QIIME 1.9.1 [23]. Quality cutoffs were performed using the Illumina standard at Q ≥ 30 for Illumina datasets followed by a paired joining. Minimum and maximum sequence lengths with the QIIME default of 200 bp and 1000 bp were used. Chimeras were removed using vsearch. Operational taxonomic units (OTU) were chosen within 97% sequence identity. SILVA database release 128 was used to assign taxonomy and align sequences [24]. Following removal of chloroplast and mitochondrial OTUs, six samples were also removed due to poor sequence yields (less than 1000 sequences per sample). Further statistical analyses were made with R version 3.4.3 [25]. The phyloseq package version 1.22.3 was used for rarefaction to even sequence depth and exclusion of singletons. Alpha diversity indices for Observed, Chao1, Shannon and Simpson metrics as well as beta diversity indices for weighted and unweighted unifrac were also calculated using the phyloseq package. P-values were calculated with ANOVA for alpha diversity and ADONIS for beta diversity with the package vegan, version 2.4–6 [26]. The package coin version 1.2–2 was used to compute differences in relative abundances on different taxonomic levels for the given time points and treatments using a Kruskal-Wallis-test for all time points, followed by a post hoc two-sided Wilcoxon-Mann-Whitney test for unpaired and non-normally distributed samples in a 10,000 fold Monte-Carlo simulation to compute the p-values between different sample groups. All p-values resulting from statistical analyses were corrected using a false discovery rate (FDR) correction for multiple testing [27].

Sequences generated and analyzed during this study are accessible at the European Nucleotide Archive (ENA) under the accession number PRJEB32559. Other datasets are available from the corresponding author on reasonable request.

## Results

### Validation of endometriosis model

The inspection of the abdomen of endometriosis mice and sham-transplanted control animals at the end of the in vivo experiments did not show any signs of bacterial infection, ascites or peritonitis. The transplantation of uterine tissue fragments resulted in the establishment of endometriotic lesions within the peritoneal cavity of all analyzed mice. However, not all fragments successfully engrafted during the 21-day observation period, as indicated by a final take rate of 23.8 ± 4.2%. The newly developed endometriotic lesions exhibited a size of 5.6 ± 1.2 mm^2^ and were particularly localized at the abdominal wall (Fig 1B) and the visceral adipose tissue around the ovary and the bladder. Moreover, a few lesions fused intensely with the wall of the small intestine. Due to their bright GFP signal, they could be easily detected in blue light epi-illumination within the surrounding GFP^-^ host tissue (Fig 1C). This approach guaranteed that no lesion was overlooked during the final inspection of the peritoneal cavity. Additional histological analyses revealed that all endometriotic lesions exhibited a typical histomorphology with cyst-like dilated endometrial glands, which were surrounded by a well vascularized endometrial stroma (Fig 1D–1G).

### Analysis of gut microbiota

Bacterial 16S rRNA genes were successfully PCR-amplified from the DNA extracts of all obtained mice fecal samples. The amplicon sequencing dataset yielded 6,315,636 partial bacterial 16S rRNA gene sequences with a mean of 150,372 sequences per sample (min: 1,408; max: 721,076 sequences). Following exclusion of singletons and rarification to even depth, 3703 OTUs affiliated with 101 genera, 35 families, 19 orders, 17 classes and 8 phyla were identified.

**Fig 2:**
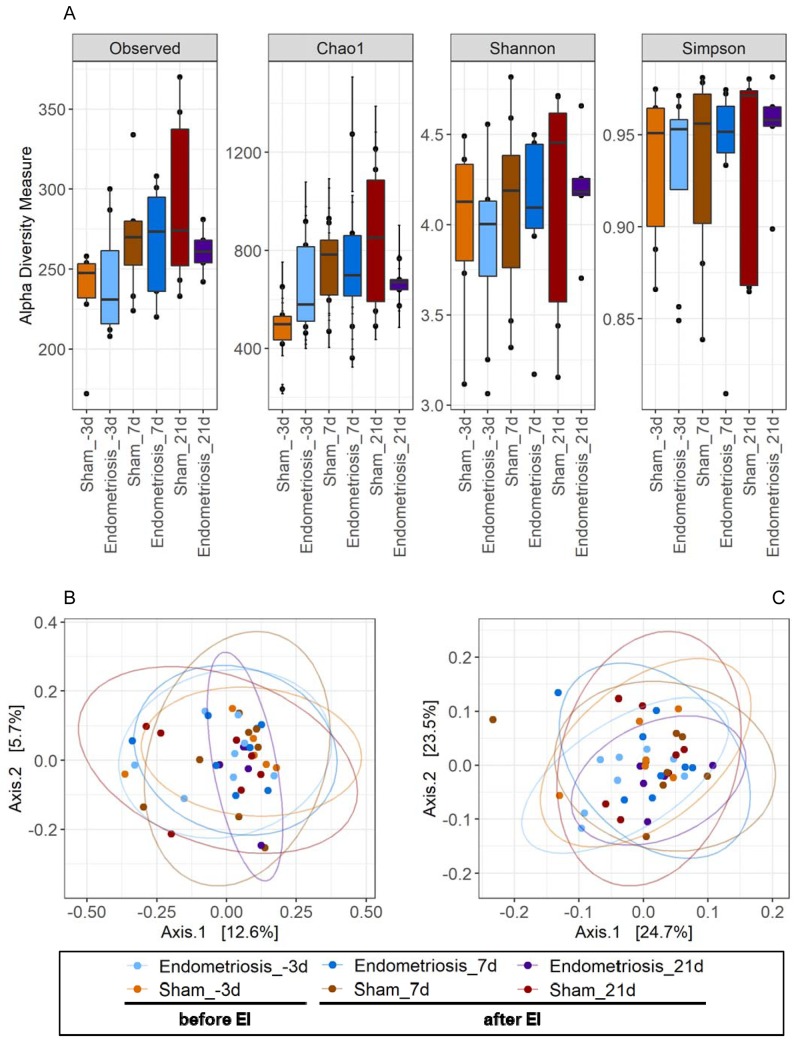
Alpha- and beta-diversity analyses. Alpha- and beta-diversity plots to visualize the difference in bacterial microbiota structure between endometriosis and sham (control) groups 3 days before the induction of endometriosis (before EI; endometriosis: n = 8, sham: n = 6) as well as on days 7 (endometriosis: n = 8, sham: n = 8) and 21 (endometriosis: n = 5, sham: n = 7) after the induction of endometriosis (after EI). Shown are alpha- diversity measures with the most common indices (A) and PCoA plots showing the beta-diversity with unweighted (B) and weighted (C) unifrac measures. Box plots (A) show median as well as lower and upper quartiles. Whiskers represent minimum and maximum spread. PCoA plots (B and C) show dimensions with the highest differences and normal confidence ellipses for the sample sets.

**Fig 3:**
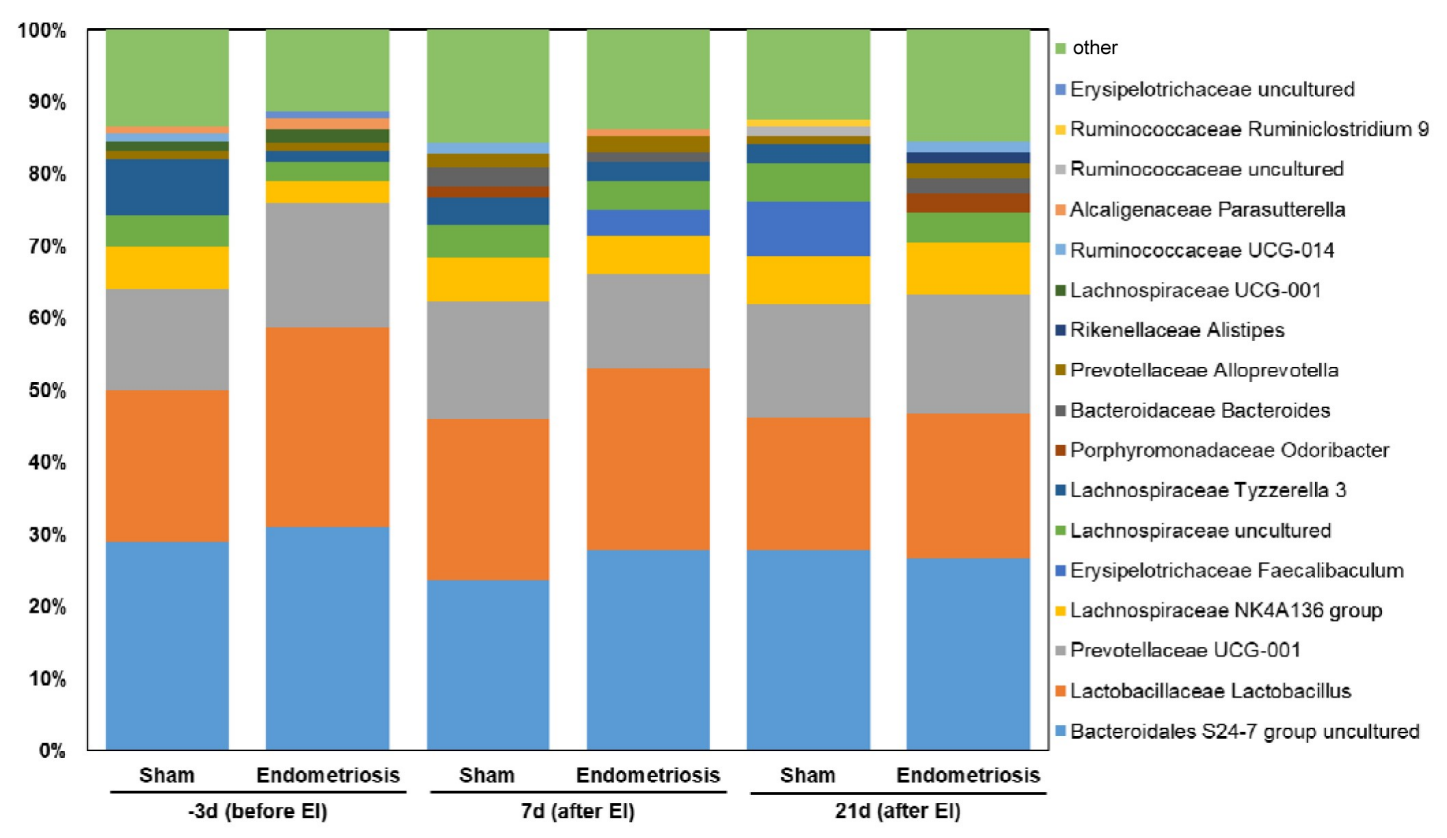
Fecal bacterial microbiota composition. Relative abundances of the 10 relatively most abundant bacterial genera in endometriosis and sham (control) groups 3 days before the induction of endometriosis (before EI; endometriosis: n = 8, sham: n = 6) as well as on days 7 (endometriosis: n = 8, sham: n = 8) and 21 (endometriosis: n = 5, sham: n = 7) after the induction of endometriosis (after EI). The genera are sorted by ascending order of relative abundances.

Alpha- and beta-diversity analyses did not reveal any significant effect of endometriosis induction on the composition of the bacterial microbiota (Fig 2), which actually remained very stable during the whole course of the experiment. More detailed analyses of the fecal bacterial microbiota composition revealed a highly diverse community with *Bacteroidales* S24-7 group, *Lactobacillus*, *Prevotellaceae* UCG-001 group and *Lachnospiraceae* NK4A136 group as the genera with the highest relative abundances (Fig 3). However, no significant differences between the different sample sets were observed, neither on genus nor on family level. P-values and FDR corrected p-values for the bacterial groups displayed in Fig 3 as well as alpha- and beta-diversity are available in the S1 Dataset of the supporting information to this article.

## Discussion

Endometriosis does not spontaneously occur in mice and other rodents, because in contrast to humans and non-human primates, these animals do not menstruate. Nonetheless, the induction of endometriosis-like lesions in rodents by transplantation of uterine tissue into the abdominal cavity is a well-established and commonly accepted approach to study various mechanisms in the pathophysiology of this complex human disease [28]. Recently, two studies suggested that the induction of endometriosis in mice leads to changes in the composition of their gut microbiota [15, 16]. This is an interesting finding considering the possibility that the detection of dysbiosis may be used in the future as a novel approach for the non-invasive diagnosis of endometriosis. However, the reported study results were quite discrepant. Chadchan et al. [15] found an altered microbial diversity in guts of endometriosis mice with a higher abundance of *Bacteroidetes* and lower abundance of *Firmicutes* already 21 days after endometriosis induction. In contrast, Yuan et al. [16] did not detect any differences in the gut microbiota of endometriosis and sham mice before 42 days. Moreover, the latter also reported alterations of *Bifidobacterium* in mice with endometriosis. These contradictory findings may be explained by numerous differences in the used experimental settings, such as the origin and diet of mice or the way of inducing endometriotic lesions. In addition, they nicely demonstrate the importance of adding new data to this very young field of endometriosis research in order to generate a more robust basis for the establishment of novel microbiota-based diagnostic tools.

Accordingly, we herein analyzed the effect of newly developing endometriotic lesions on the composition of the gut microbiota in mice. For this purpose, we chose rather early observation time points, i.e. 7 and 21 days after endometriosis induction, because previous studies indicated that particularly in this early phase uterine tissue grafts are characterized by a high angiogenic and proliferative activity of their endometrial stroma [17]. Moreover, we freely transplanted uterine tissue grafts of equal size from GFP^+^ donor animals into the peritoneal cavity of GFP^-^ wild-type mice. This standardized GFP^+^/GFP^-^ cross-over design markedly facilitated the identification of successfully engrafted endometriotic lesions during the final inspection of the peritoneal cavity and, thus, guaranteed that fecal samples were only analyzed from mice with comparable disease-mimicking conditions. Thereby, it should be considered that the take rate was only ~24% in our study. However, such a low take rate is typical for the induction of endometriotic lesions by random inoculation of uterine tissue into the peritoneal cavity [28]. Furthermore, it should be noted that we used mice, which received only an intraperitoneal injection of physiological saline solution, as sham-transplanted controls. This did not mimic the contamination of the abdominal cavity with ectopic tissue. Hence, we cannot rule out that the results of our microbiota analysis are primarily determined by the tissue transplantation process itself and not by uterine tissue-specific effects. To address this issue in future studies, it may be recommended to transplant a completely other type of tissue, such as lung or gut, into the abdominal cavity as an additional control. However, one should be aware that those tissues contain specific cell populations, such as alveolar macrophages or dendritic cells, and, thus, may be capable of exerting additional individual effects on the gut microbiota.

Our cultivation-independent, 16S rRNA gene-amplicon based sequencing analysis of the fecal bacterial microbiota revealed a highly diverse community composition, which—based on the identified bacterial taxa and considering the usual range of housing facility-based differences—can be regarded as typical for the intestinal tract of healthy mice [29, 30]. In line with the study of Yuan et al. [16], we found that the bacterial community composition seemed to remain unaffected by the induction of endometriosis over the 21-day experiment, indicating no signs of intestinal dysbiosis in this early phase of lesion formation. In this context, it should be emphasized that our data analysis and applied statistical analyses were quite strict. We removed singleton OTUs in addition to chimeras in order to lower the number of potential sequencing errors and to improve the quality of the data set. Removal of singletons lowers the overall alpha- and beta-diversity [31]. In addition, we used a false discovery rate correction (FDR) for all p-values in order to reduce the probability of reporting false positive significances due to multiple testing [27]. To reduce a possible type-I-error, we used the rather conservative Kruskal-Wallis-test and the Wilcoxon-Mann-Whitney-test, even though the design was originally a repeated measure design. Both quality steps, singleton removal and FDR correction, as well as the more rigid test, may have contributed to the lack of significant differences between endometriosis and sham-operated control animals in the present study. Moreover, it cannot be ruled out that more powerful studies including more test animals and a higher sequencing depth might unravel significant changes in gut microbiota composition also in the early phase of endometriosis.

Nevertheless, our data allow careful speculation that there might be no pronounced alterations of the gut microbiota in the very early phase of lesion formation. Yuan et al. [16] indicate that such alterations may be rather detected in later stages of the disease, caused by a long-lasting intestinal disturbance and damage. Their experimental findings are supported by the recently published Endobiota study, which compared for the first time the gut microbiota between women with stage 3/4 endometriosis and healthy controls [32]. Of interest, this study showed *Escherichia* and *Shigella* were more dominant in the gut microbiota of endometriosis patients when compared to controls. In light of the fact that endometriosis patients often consult a physician after longer time frames of suffering from unspecific symptoms, it may not be a problem that endometriosis-induced alterations of the gut microbiota are also only detected in later stages of endometriotic lesion formation.

Finally, it should be considered that the microbiological data presented here and in previous experimental studies [15, 16] are DNA-based, i.e. they do not allow discriminating active from less active or even dead bacterial taxa. However, it may be speculated that the induction of endometriosis rather affects microbial activity, including gene expression and metabolism, than the overall community structure. Hence, follow-up studies should be additionally based on the analysis of microbial RNA and/or metabolites [33] in order to better address changes in microbial functionality under the influence of endometriosis.

## Supporting information

S1 Dataset P-values and FDR corrected p-values for the bacterial groups displayed in Fig 3 as well as alpha- and beta-diversity. (XLSX)
